# Childhood hematologic cancer and residential proximity to oil and gas development

**DOI:** 10.1371/journal.pone.0170423

**Published:** 2017-02-15

**Authors:** Lisa M. McKenzie, William B. Allshouse, Tim E. Byers, Edward J. Bedrick, Berrin Serdar, John L. Adgate

**Affiliations:** 1 Department of Environmental and Occupational Health, Colorado School of Public Health, University of Colorado Anschutz Campus, Aurora, Colorado, United States of America; 2 Department of Epidemiology, Colorado School of Public Health, University of Colorado Anschutz Campus, Aurora, Colorado, United States of America; 3 Epidemiology and Biostatistics Department, Mel &Enid Zuckerman College of Public Health, University of Arizona, Tucson, Arizona, United States of America; Stony Brook University, Graduate Program in Public Health, UNITED STATES

## Abstract

**Background:**

Oil and gas development emits known hematological carcinogens, such as benzene, and increasingly occurs in residential areas. We explored whether residential proximity to oil and gas development was associated with risk for hematologic cancers using a registry-based case-control study design.

**Methods:**

Participants were 0–24 years old, living in rural Colorado, and diagnosed with cancer between 2001–2013. For each child in our study, we calculated inverse distance weighted (IDW) oil and gas well counts within a 16.1-kilometer radius of residence at cancer diagnosis for each year in a 10 year latency period to estimate density of oil and gas development. Logistic regression, adjusted for age, race, gender, income, and elevation was used to estimate associations across IDW well count tertiles for 87 acute lymphocytic leukemia (ALL) cases and 50 non-Hodgkin lymphoma (NHL) cases, compared to 528 controls with non-hematologic cancers.

**Findings:**

Overall, ALL cases 0–24 years old were more likely to live in the highest IDW well count tertiles compared to controls, but findings differed substantially by age. For ages 5–24, ALL cases were 4.3 times as likely to live in the highest tertile, compared to controls (95% CI: 1.1 to 16), with a monotonic increase in risk across tertiles (trend p-value = 0.035). Further adjustment for year of diagnosis increased the association. No association was found between ALL for children aged 0–4 years or NHL and IDW well counts. While our study benefited from the ability to select cases and controls from the same population, use of cancer-controls, the limited number of ALL and NHL cases, and aggregation of ages into five year ranges, may have biased our associations toward the null. In addition, absence of information on O&G well activities, meteorology, and topography likely reduced temporal and spatial specificity in IDW well counts.

**Conclusion:**

Because oil and gas development has potential to expose a large population to known hematologic carcinogens, further study is clearly needed to substantiate both our positive and negative findings. Future studies should incorporate information on oil and gas development activities and production levels, as well as levels of specific pollutants of interest (e.g. benzene) near homes, schools, and day care centers; provide age-specific residential histories; compare cases to controls without cancer; and address other potential confounders, and environmental stressors.

## Introduction

Among U.S. children ages 0–14, acute lymphocytic leukemia (ALL) is the most commonly diagnosed cancer, and non-Hodgkin lymphoma (NHL) is the most common lymphoma [1]. While ALL and NHL mortality rates in U.S. children are declining due to improved treatment, ALL and NHL incidence rates have increased by about 1% and 0.6% per year, respectively, between 2000 and 2010 [2, 3].

A number of factors, including genetic predisposition and susceptibility, as well as environmental factors, come together in the development of childhood cancers through a two step process, the first of which likely occurs in utero [4]. Environmental factors that may be associated with ALL include in-utero and postnatal exposures to vehicle exhaust fumes [5, 6], polycyclic aromatic hydrocarbons [7,8], and chemicals including benzene and other hydrocarbons [8–11]. Environmental factors that may be associated with NHL include benzene exposures [12].

U.S. oil and gas development has grown rapidly over the past 15 years. This industrial activity, which includes drilling, hydraulic fracturing, and production, has the potential to emit chemicals that may be associated with childhood ALL and/or NHL, including benzene and other hydrocarbons, polycyclic aromatic hydrocarbons, and diesel exhaust, into the air and water [13–18]. The use of hydraulic fracturing and horizontal drilling has facilitated extraction of petroleum reserves from shale and other tight formations, resulting in an extensive de-centralized dispersion of oil and gas wells and associated facilities across populated areas [19]. This has the potential to expose a large populaton to oil and gas development related pollutants. It is estimated that over 15.3 million Americans now live within 1.6 kilometers of an oil and gas well drilled since the year 2000 [19]. In Colorado’s most intensive areas of oil and gas development, there may be hundreds of oil and gas wells within 1.6 kilometers of a home. The existing literature indicates that populations living in areas with oil and gas development may be at an increased risk for health effects, including cancers such as ALL and NHL, resulting from these exposures [20].

Previous ecological studies on the link between childhood cancer and oil and gas development are inconclusive because they aggregated cancer outcomes and exposures and did not consider latency periods and differentiate between types of leukemias [21, 22]. This analysis addresses these limitations by comparing individual-level residence of incident cancer cases to geocoded oil and gas well locations over a 10-year latency period using a registry based case-control approach to explore if children living near oil and gas development may be at higher risk for childhood ALL and NHL compared to children diagnosed with non-hematologic malignancies and to inform the design of more comprehensive studies.

## Methods

### Study population

Childhood incidence cancer data for children diagnosed with cancer between 2001 and 2013 were obtained from the Colorado Central Cancer Registry (CCCR) [23] held at the Colorado Department of Public Health and Environment (CDPHE). Our study population consisted of 975 children in the registry diagnosed with cancer at ages 0–24 years, and residing in rural Colorado at the time of diagnosis. Subjects aged 15–24 years were included in the study to account for a possible 10 year latency period between childhood exposures before the age of 15 and onset of cancer [24–27]. We restricted analysis to cancers occurring from 2001–2013 to focus our analysis on growth of hydraulic fracturing and/or directional drilling, which expanded rapidly in Colorado beginning around the year 2000 [28]. This rapid expansion resulted in a twofold increase in active oil and gas wells in Colorado between 2000 and 2013; by 2013, Colorado had more than 51,000 oil and gas wells. We restricted the study area to rural areas and towns with populations of <50,000 in 57 counties to reduce potential for exposure to other pollution sources, such as traffic, and other industrial sources. Geocoded precise addresses were not available for 232 children (including 17 and 15 children diagnosed with ALL and NHL, respectively), so those children were excluded from the study (e.g., addresses not listed, specified as rural routes, or only as post office boxes). Using the final study group of 743 children, we conducted two separate registry-based case control studies to explore associations between childhood hematologic cancers and density of oil and gas development operations around the child’s residence. Cases were children with ALL or NHL. Because we only had access to records in the CCCR for this exploratory study, controls were all children registered in the CCCR with a non-hematologic cancer, as has been done in previous registry based studies [29–32].

### Exposure to oil and gas wells

We used information available in the publically accessible Colorado Oil and Gas Information System (COGIS) [33] to build a geocoded dataset with latitude and longitude coordinates of oil and gas wells in rural Colorado, and determined whether or not each well was active for each year between 1991 and 2013, as defined by the Colorado Oil and Gas Conservation Commission (COGCC) [28] (see S1 Table). The COGCC considers oil and gas wells to be active between the spud-in and abandon dates. “Spud-in” is the operation of drilling the first part of a new well; “abandon” is the permanent plugging of a well [28]. No spud-date is recorded for 28% of oil and gas wells in the COGIS. If a spud-in date was not available for a well, we used the earliest date available from other oil and gas activities which follow the spud-in (e.g., completion, first production, and treatment dates) to designate the beginning of the active well period. We provided this dataset of geocoded well locations to CDPHE staff.

To protect the identity of the children in the study, CDPHE staff linked the well locations in the dataset we provided to each child’s address in the registry. Geocoded residential addresses at time of cancer diagnosis were linked to active well locations in the year of diagnosis, as well as active well locations in each of the 10 years preceding the cancer diagnosis. Distance of each residence from all active oil and gas wells within a 16.1-kilometer radius was then computed using spherically-adjusted straight line distances, to account for the Earth’s curvature. Ages were truncated into five year ranges (0–4, 5–9, 10–14, 15–19, and 20–24 years) to further protect identity. The CDPHE staff then provided the analyst (LMM) with a de-identified dataset, containing subject diagnosis, age group, gender, race/ethnicity, income quintile at zip code level, residential elevation, maternal smoking history (from linkages with birth certificates), and distance to each active oil and gas well within a 16.1-kilometer radius.

**Fig 1:**
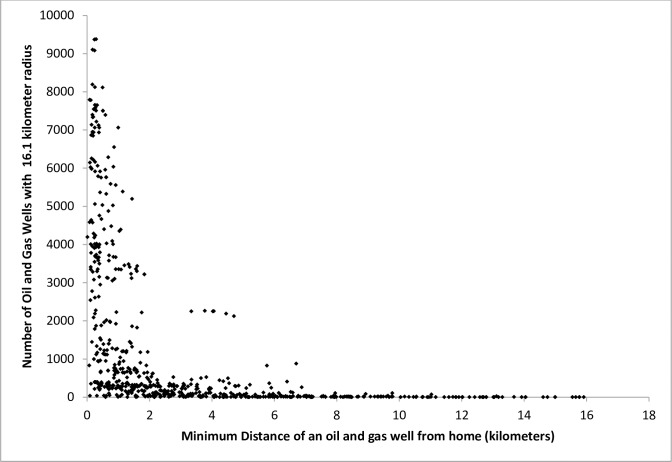
Number of oil and gas wells in 16.1-kilometer radius from a child’s home versus the minimum distance of an oil and gas well from the child’s home for children with at least one oil and gas well within the 16.1-kilometer radius.

Because the number of wells is not associated with distance of the nearest well in the 16.1-kilometer radius around a child’s home (Fig 1), we used an inverse distance weighted (IDW) approach to estimate the density of oil and gas wells surrounding a child’s home. The IDW approach is commonly used to estimate individual air pollutant exposures from multiple fixed locations [34–36]. Annual IDW well counts account for the number of active wells within the 16.1-kilometer radius [36, 37] of the residence in a specific year as well as distance of each well from the residence, giving greater weight to wells closest to the residence. For example, an IDW well count of 78.1 wells per kilometer could be computed from 125 wells each located 1.6 kilometers from the maternal residence or 25 wells each located 0.32 kilometers from the child’s residence.

Based on the latency period between exposure to benzene and diagnosis of leukemia of 1 to 10 years [24–27], we computed weighted average IDW well counts (referred to herein as IDW well count) over a 10 year period to capture IDW wells counts during the antenatal period and childhood. Because we had age ranges rather than specific ages, we averaged the annual IDW well count over a temporal range that would capture the latency period for five age categories as follows. For children aged 0–4 years, annual IDW well counts for 1–5 years prior to diagnosis were averaged over 5 years. For children aged 5–9, 10–14 and 15–19 years, annual IDW well counts for 1–10 years prior to diagnosis were averaged over 10 years. For young adults 20–24 years, annual IDW well counts 6–10 years prior to diagnosis were averaged over 5 years to account for childhood exposures (i.e., occurring prior to age fifteen). If no wells existed within 16.1-kilometers of a child’s home during the latency period for each age range, the weighted average IDW well count was 0. The final distribution of the non-zero IDW well counts was then divided into tertiles (low, medium, and high) for subsequent logistic regression. Each tertile was compared to the referent group of an IDW well count of zero.

### Analysis

Childhood cancer outcomes considered for analysis were ALL (first primary tumor, includes Burkitt leukemia) (87 cases), and NHL nodal and extra-nodal (includes Burkitt and lymphoblastic lymphomas regardless of bone marrow involvement) (50 cases). The number of cases for other types of leukemia (<15 cases for each type including acute myeloid leukemia, and 26 cases combined) was too small to analyze individually. Cases diagnosed with other leukemia types (n = 26) and those with Hodgkin lymphoma (n = 52) were excluded from the control group. Case-control analyses were conducted separately for ALL and NHL. A total of 528 controls for each of these cancer outcomes included all children reported in the CCCR with non-hematologic cancers, (Table 1).

**Table 1:** Cancer sites for children in rural Colorado 2001 to 2013^1^.

Cancer Site^2^	Frequency
**Blood and lymph system**	
-acute lymphocytic leukemia	104
-Hodgkin lymphoma	65
-non-Hodgkin lymphoma	65
-acute myeloid leukemia	18
-myeloma, other lymphocytic leukemia, chronic myeloid leukemia, other myeloid/monocytic leukemia, other acute leukemia, aleukemic,subleukemic and NOS	<5 of each
Nervous System (brain, cranial and other)	169
Endocrine System	136
**Male genitals**	
-testes	75
-penis	<5
**Skin**	
-Melanomas	76
-Other skin cancers	<5
Soft Tissue (including heart)	46
Bones and joints	42
**Female genitals**	
-Ovary	16
-Cerix Uteri	14
-Vagina, vulva, other	< 5 of each
**Urinary System**	
-kidney and renal pelvis	30
-bladder	< 5
Eye and orbit	15
**Digestive System**	
-liver	6
-esophagus, stomach, small intestine, appendix, colon and rectum, pancreas, retroperitoneum,cecum	< 5 each
**Respiratory**	
- lung and bronchus	7
-trachea, mediastinum and other respiratory; nose, nasal cavity, and middle ear; larynx	< 5 of each
Breast	8
Oral Cavity and Pharynx (tongue, salivary glands, gum, nasopharynx)	<5 of each

^1^Includes children without geocoded addresses.

^2^17 cancers were classified as miscellaneous and less than 5 were classified as invalid.

### Statistical analysis

We used logistic regression to study associations between case-control status and IDW well count tertiles. First, we estimated the crude odds ratio (OR) associated with IDW well counts tertiles. We further investigated associations by adjusting for potential confounders, as well as child covariates, based on *a priori* knowledge of their association with both exposure and outcome. We conducted tests to evaluate linear trends in binominal proportions with increasing IDW well count by treating the categorical IDW well count variable as ordinal [38]. Specifically, covariates in our analysis were age category (0–4, 5–9, 10–14, 15–19, and 20–24 years) [1,4], race/ethnicity (white Hispanic, white non-Hispanic, non-white) [1], and gender [1]. Because the age range 0–4 years and ≥5 years may respresent two distinct disease etiologies for ALL [4], we analyzed separately for children ages 0–4 years and ≥ 5 years. We adjusted for elevation of residence (< 2743 meters, ≥ 2743 meters because residential solar ultraviolet radiation exposure, which increases with elevation [39], may be associcated with ALL [40]. We also adjusted for socio-economic status by distributing zip-code level median income into quintiles (0–20, 21–40, 41–60, 61–80, and 81–100 percentiles) [5]. In a second model, we further adjusted for year of cancer diagnosis (2001–2002, 2003–2004, 2005–2006, 2007–2008, 2009–20110, 2011–2013). We did not adjust for maternal smoking during pregnancy in the primary analyses because information on maternal smoking was missing for 59% of the study population. We report estimations for each outcome associated with the IDW well count tertile (low, medium, and high) compared to no wells within 16.1-kilometers with 95% confidence intervals (CI). We considered both the statistical significance of the association for each IDW well count category as well as the trend across categories in evaluating results, using a 2-sided alpha of 0.05.

In secondary analyses, we explored reducing IDW well counts to an 8-kilometer buffer around the maternal residence, as well as analyzing the subset of subjects with information on maternal smoking and adjusting for maternal smoking during pregnancy. All statistical analyses were conducted using SAS® software version 9.3 (Cary, NC). To further protect the identity of study participants we truncated counts of less than 5 to “<5” in result tables. The Colorado Multiple Institutional Review Board reviewed and approved our specific protocol for this study as exempt from human subjects research (COMIRB Protocol 14–1978). We (the researchers) only had access to de-identified data from the Colorado Cancer Registry which had been previously collected for reasons other than research and the data we analyzed anonymously.

## Results

Geocoded addresses were missing for 24% of our study population, with a higher proportion missing for white Hispanics and young adults aged 19–24 years, as well as controls (Table 2). The ALL cases were more likely than controls to be < 10 years of age, male, a non-white race, to live at an elevation > 2743 meters, and in the highest zip code median income quintile (Table 2). The NHL cases were more likely than controls to be 5–14 years of age, male, a non-white race, and to live in the middle zip code median income quintiles (Table 2). In the final study population of 743 children with cancer, 73% resided in a home within one of the IDW well count tertiles (≥ 1 active oil and gas well in a 16.1-kilometer radius) (Table 3). The IDW well counts were higher for children living at lower elevations (< 2134 meters), among children aged 0–4 years, and for children in the high zip code median income quintiles (Table 3).

**Table 2:** Study population characteristics for children residing in rural Colorado and diagnosed with cancer between 2001 and 2013 by case control status.

	NotGeocoded^a^	Geocoded	Geocoded ALL Cases	Geocoded NHL Cases	Geocoded Controls^b^
**Number of children (n)**	232	743	87	50	528
**Race/Ethnicity (% of children)**					
White—Non-Hispanic	76	76	77	78	77
White–Hispanic	21	15	12	<10	17
Non-white	2.8	7.8	12	16	5.7
Missing	6	0.67	0	0	0.95
**Gender (% of total)**					
Male	52	52	60	68	50
**Elevation of residence (% of children)**					
< 1524 meters	NA	31	37	32	31
1524–1828 meters	NA	27	25	28	25
1829–2133 meters	NA	19	20	24	19
2134–2437 meters	NA	14	8.0	12	16
2438–2742 meters	NA	5.4	<6	<10	6.1
2743meters or higher	NA	3.9	6.9	<10	3.4
**Age range (% of children)**					
0–4 years	16	20	45	10	18
5–9 years	11	9.7	26	18	6.4
10–14 years	12	12	14	18	12
15–19 years^c^	15	23	11	22	24
20–24 years^d^	46	35	<6	32	39
**Zip code median income quintiles (% of children)**					
0–20	7.0	9.2	9.2	<10	9.2
21–40	21	25	21	18	26
41–60	22	14	14	26	14
61–80	25	27	25	32	28
81–100	24	24	31	20	24
**Year of Diagnosis (% of children)**					
2001–2002	22	13	10	<10	13
2003–2004	16	16	18	22	15
2005–2006	16	14	15	14	13
2007–2008	12	16	20	12	16
2009–2010	13	18	14	24	19
2011–2013	20	23	23	18	23

^a^Geocoded addresses were not 0061vailable (e.g., addresses not listed, or specified as rural routes or post office boxes) for 16% of ALL cases, 23% of NHL cases and 28% of controls.

^b^Diagnosed with non-hematologic cancer (See Table 1).

^c^Children from 15 to 19 years were included in the study to account for a latency period of 10 years between exposure and onset of cancer.

^d^Children from 20–24 years were included in the study to account for a latency period of 10 years between exposure and onset of cancer.

NA = not available.

**Table 3:** Study population characteristics for children residing in rural Colorado with a geocoded address and diagnosed with cancer between 2001 and 2013 by exposure group.

	No Wells within 16.1Kilometers	Exposure Tertiles		
		Low^a^	Medium^a^	High^a^
**Number of children (n)**	199	180	179	185
**Race/Ethnicity (% of children)**				
White—Non-Hispanic	75	78	79	74
White–Hispanic	17	14	13	17
Non-white	8.0	6.7	7.8	8.6
Missing	0	1.1	< 1	1.1
**Gender (% of children)**				
Male	55	45	55	56
**Elevation of residence (% of children)**				
< 1524 meters	10	35	37	45
1524–1828 meters	16	19	33	39
1829–2133 meters	11	31	26	11
2134–2437 meters	32	13	3.9	4.9
2438–2742 meters	18	2.2	0.56	0
2743meters or higher	15	0	0	0
**Age range (% of children)**				
0–4 years	14	21	18	26
5–9 years	9.1	6.1	12	11
10–14 years	11	11	16	13
15–19 years^b^	26	24	21	19
20–24 years^c^	41	37	32	30
**Zip code median income quintiles (% of children)**				
0–20	17	10	7.2	0
21–40	33	28	35	5.4
41–60	9.1	11	28	8.1
61–80	19	29	17	44
81–100	20	22	12	43
**Year of Diagnosis (% of children)**				
2001–2002	11	17	12	12
2003–2004	13	18	17	17
2005–2006	17	8.9	17	13
2007–2008	18	18	14	15
2009–2010	16	21	19	16
2011–2013	26	18	21	26

^a^ Low = first tertile, < 4.96 wells per 1.6 kilometers, medium = second tertile, 4.96 to 33.6 wells per 1.6 kilometers high = third tertile, more than 33.6 wells per 1.6 kilometers.

^b^Children from 15 to 19 years were included in the study to account for a latency period of 10 years between exposure and onset of cancer.

^c^Children from 20 to 24 years were included in the study to account for a latency period of 10 years between exposure and onset of cancer.

Both crude and adjusted estimates indicate an increase in odds of living near oil and gas development at the time of cancer diagnosis, as represented by IDW well counts, in children diagnosed with ALL (Table 4; see S2 and S3 Tables for the full logistic models). Overall, children aged 0–24 years diagnosed with ALL were more than 2 times as likely as controls to live in areas with active oil and gas wells within 16.1-kilometers of their residence during the latency period (p for trend = 0.22) after adjusting for age, race, gender, income, and elevation in model 1. Children aged 5–24 years diagnosed with ALL were 4.3 (95% CI: 1.1 to 16) times as likely as controls to live in the highest IDW well count tertile, and a monotonic increase across the IDW well count tertiles was observed (p for trend = 0.035) (Table 4). Further adjustment for year of diagnosis in model 2 resulted in slightly larger associations. We observed no statistically significant association between ALL and proximity to oil and gas development in our analysis of ALL for children ages 0–4 years in either model.

**Table 4:** Association between annual inverse distance weighted well count within 16.1-kilometer radius of residence at diagnosis averaged over exposure period and acute lymphocytic leukemia (ALL).

Inverse Distance Weighted Well Count^a^	0 Wells within 16.1 Kilometers	Low^a^	Medium^a^	High^a^	P-value trend tests^b^
***Total Study Population (0 to 24 years)***					
Cases (N)	15 (9.2%)	21 (14%)	26 (18%)	25 (16%)	
Controls (N)	147	132	119	130	
Crude OR	1.0	1.6 (0.77, 3.1)	2.1 (1.1, 4.2)	1.9 (0.95, 3.7)	
Model 1 Adjusted OR (95% CI)^c^	1.0	2.3 (0.94, 5.5)	2.6 (1.1, 6.3)	1.9 (0.78, 4.8)	0.22
Model 2 Adjusted OR (95% CI)^d^	1.0	2.5 (1.0, 6.1)	2.8 (1.2, 6.8)	2.0 (0.80, 5.0)	0.21
***5 to 24 Years***					
Cases (N)	8 (5.9%)	9 (7.7%)	15 (13%)	16 (14%)	
Controls (N)	128	108	99	96	
Crude OR	1.0	1.3 (0.50, 3.6)	2.4 (0.99, 5.9)	2.7 (1.1, 6.5)	
Model 1 Adjusted OR (95% CI)^c^	1.0	2.9 (0.80, 11)	3.4 (0.99, 12)	4.3 (1.1, 16)	0.035
Model 2 Adjusted OR (95% CI)^d^	1.0	3.2 (0.84, 13)	3.6 (1.0, 13)	4.6 (1.2, 18)	0.032
***0 to 4 Years***					
Cases (N)	7 (26%)	12 (33%)	11 (35%)	9 (21%)	
Controls (N)	19	24	20	34	
Crude OR	1.0	1.4 (0.45, 4.1)	1.5 (0.48, 4.7)	0.72 (0.23, 2.2)	
Model 1Adjusted OR (95% CI)^e^	1.0	1.7 (0.44, 6.5)	2.3 (0.57, 9.4)	0.73 (0.18, 3.0)	0.50
Model 2 Adjusted OR (95% CI)^f^	1.0	1.5 (0.38, 5.9)	2.3 (0.58, 9.4)	0.51(0.12, 2.2)	0.31

^a^ All age groups: low = first tertile, < 4.9 wells per 1.6 kilometers, medium = second tertile, 4.9 to 33.6 wells per 1.6 kilometers, high = third tertile, more than 33.6 wells per 1.6 kilometers.

^b^Trend tests performed by treating categorical inverse-distance well count as an ordinal.

^c^Adjusted for age, race, gender, socioeconomic status, and elevation.

^d^Adjusted for age, race, gender, socioeconomic status, elevation, and year of diagnosis.

^e^Adjusted for race, gender, socioeconomic status, and elevation.

^f^Adjusted for race, gender, socioeconomic status, elevation, and year of diagnosis.

In secondary analyses using an 8-kilometer radius, results for cases of ALL ages 5–24 years and ages 0–4 years were similar to the primary analysis (S4 Table). In secondary analyses on the subset of children for whom information on maternal smoking was available, we observed similar associations to our primary analysis, with little evidence for confounding by maternal smoking history (S5 Table).

We observed no statistically significant associations between density of oil and gas development and NHL in either model, based on trend analysis across categorical IDW well counts (Table 5) (see S6 and S7 Tables for the full logistic models). The observed outcomes for NHL from the analyses using an 8-kilometer radius or in the subsets of subjects with information on maternal smoking did not change the inferences drawn from the primary analysis (see S8 and S9 Tables).

**Table 5:** Association between annual inverse distance weighted well count within 16.1 kilometer radius of residence at diagnosis averaged over the exposure period and non-Hodgkins lymphoma (NHL).

Inverse Distance Weighted Well Count	0 Wells within 16.1 Kilometers	Low^a^	Medium^a^	High^a^	P-value for trend tests^b^
Cases (N)	13 (8.1%)	13 (9.0%)	11 (8.5%)	13 (9.0%)	
Controls (N)	147	132	119	130	
Crude OR	1.0	1.1 (0.50, 2.5)	1.0 (0.45, 2.4)	1.1 (0.51, 2.5)	
Model 1Adjusted OR (95% CI)^c^	1.0	1.2 (0.51, 2.9)	0.71 (0.28, 1.8)	1.0 (0.41, 2.6)	0.89
Model 2 Adjusted OR (95% CI)^d^	1.0	1.2 (0.49, 2.8)	0.63 (0.25, 1.6)	0.99 (0.39, 2.5)	0.61

^a^ low = first tertile, < 4.9 wells per 1.6 kilometers, medium = second tertile, 4.9 to 33.6 wells per 1.6 kilometers, high = third tertile, more than 33.6 wells per 1.6 kilometers.

^b^Trend testsperformed by treating categorical inverse-distance well count as an ordinal.

^c^Adjusted for age, race, gender, socioeconomic status and elevation.

^d^Adjusted for age, race, gender, socioeconomic status, elevation, and year of diagnosis.

## Discussion

In this registry-based case-control study, we found that children aged 5–24 years diagnosed with ALL were 3–4 times as likely to live in areas with active oil and gas wells as were children diagnosed with non-hematologic cancers, and the association between ALL and residential density of oil and gas wells increased monotonically from the lowest to highest IDW well count categories after adjusting for age, race, gender, socioeconomic status, and elevation. Further adjustment for year of cancer diagnosis resulted in a slightly larger association in children aged 5–24 years. We did not observe an association between ALL and density of active oil and gas wells in children aged 0–4 years. We found no indication of an association between NHL and density of active oil and gas wells.

In model 2, we categorized year of cancer diagnosis into six categories each containing two to three diagnosis years (Table 2). We note that if diagnosis year is not aggregated into six categories, the association in children aged 5–24 years attenuates to 3.6 (CI: 0.93, 14) and the p-value for the trend is 0.056 (S10 Table). Aggregating diagnosis year results in 13 categories, several of which have <5 observations. In a small data set such as the data set for the 5–24 year age group, minor changes to subsets of the data with < 5 observations can easily lead to p-values switching from 0.03 to 0.06 or vice-versa. We selected the model with diagnosis year categorized into six categories because it is the more robust model.

Our analysis addressed the limitations of previous studies. The case-control design allowed us to evaluate individual outcomes and IDW well counts; and we considered a 1–10 year latency period and one specific type of childhood leukemia, rather that grouping all leukemias, in our analysis. An ecological study on childhood cancer incidence associated with hydraulic fracturing sites in Pennsylvania found no differences between childhood leukemia incidence before and after hydraulic fracturing began in several Pennylvania counties [21]. That study aggregated all leukemia cases at the county level, and did not fully consider a latency period [41]. An ecological study in Texas aggregated cases at the zip-code level, finding higher childhood ALL incidence rates than expected in two zip codes with oil and gas development, but that difference was not statistically significant with only 11 cases [22].

It is thought that less than 5% of leukemia is attributed to only inherited genetic predisposition [4]; that genetic suscectibility and environmental factors combine in the development of ALL [4]; and that the etiology of infant ALL (<1 year) may differ from that of childhood ALL [42, 43]. Most infant ALL may develop through a one step process, the intiation of preleukemic clone involving MLL gene rearrangement at 11q23 in utero [4, 42–44]. Childhood ALL is thought to develop through a two step process [4, 43, 44], in which the first step also occurs in utero, with initiation of a preleukemic clone most often involving other gene rearrangments (e.g., TEL_AML rearrangements) [4, 42, 45]. The role played by chance versus other factors in the generation of in-utero preleukmeic clones in both infant and childhood ALL is unclear [4]. The second step for childhood ALL, which possibly involves an interaction between genetic suscectibiltiy and environmental factors post-utero, converts the preleukemic clone to leukemia [4,42, 46]. This two step process may partially explain why we observed stronger associations between ALL and density of oil and gas development when we evaluated children ages 5 to 24 years than among those ages 0–4 years, which includes infant ALL.

One possible environmental risk factor for childhood ALL that is associated with oil and gas development is exposure to benzene and other petroleum hydrocarbons. Ambient air benzene levels in Colorado areas with active oil and gas development ranged from 0.03–22 parts per billion by volume (ppbv). Median benzene concentrations ranged from 0.212–0.757 ppbv which are greater than the Environmental Protection Agency’s (EPA) risk based screening level (RBSL) of 0.102 ppbv for benzene in residential air [18, 20, 47–50]. Benzene concentrations in groundwater samples collected at oil and gas development sites in northeastern Colorado associated with surface spills range from less than 1–12,000 parts per billion (ppb), with a median of 1.5 ppb [16], which is greater than EPA’s RBSL of 0.45 ppb for benzene in tap water [50]. It is important to note that EPA’s RBSLs for benzene are based on cancer concerns.

Benzene is a well established cause of acute myeloid leukemia in adults [51]. Studies of benzene exposures and acute leukemias in children are limited and less conclusive. An ecological study in Texas reported that census tracts with the highest benzene levels (1.6 ppbv) had elevated rates of childhood ALL [11]. A case-control study in France reported that children aged 0–14 years living in a home adjoining to a gas station or repair garage were at increased odds of ALL [10]. Another case-control study in California reported elevated odds of ALL in children aged 0–5 years exposed to ambient levels of benzene and xylenes in the their third trimester of pregnancy [8]. However, a recent study in France found no assocation between benzene emissions from heavy traffic and ALL [52].

Our study benefited from the ability to select cases and controls from the same population. Because all our data were obtained from the CCCR, neither recall bias nor measurement bias affected our results. The possibility of random errors does exist in the CCCR, but in our judgment these are unlikely to affect our findings.

Geocoded addresses were missing for 27% of our study population, with a higher proportion of missing addresses for white Hispanics and young adults aged 19–24 years, as well as controls. These groups are more likely to include migrant workers, college students, and undocumented residents, so our results may not be representative of these groups.

Because ALL and NHL are rare diseases, there were only a small number of ALL and NHL cases in rural Colorado between 2001 and 2013. This limited number of cases affected the stability of our associations, as evidenced by wide confidence intervals, particularly for ALL in the 5–24 year and 0–4 year subsets and in the NHL analyses. Our controls were children diagnosed with a non-hematologic cancer, which may share some risk factors with NHL and ALL, including exposure to hazardous air pollutants associated with oil and gas development. This may have biased our associations toward the null [29, 53]. We note that between 2000 and 2013: (1) in rural Colorado counties, the mean annual incidence of ALL increased more rapidly than the total cancer incidence with an annual mean increase of 2.9% per year compared to 0.51% per year; and (2) in urban Colorado counties, the mean annual incidence of ALL decreased by 0.5% [23]. In addition, our observed 5.9% proportion of ALL to other cancers in the unexposed group (no wells within 16.1 km) is similar to rural population proportions populations in the neighboring states of Nebraska (6.1% and Utah (4.6%) [54]. Finally, 82% of the children in the 0–4 year age group resided in a home within one of the IDW well count tertiles (one or more active oil and gas wells in a 16.1-kilometer radius) compared to 71% of children in the 5–24 year age groups.

Data on covariates obtained from the CCCR were limited to basic demographic information and median incomes at the zip code level. Our inability to adjust for early common infections, nutrition, family history of neoplasms, water source, proximity to other pollutants, and daycare attendance, as well as individual income, may have resulted in residual confounding. Meta analyses indicate that children with ALL and NHL are 10% and 22%, respectively, more likely than controls to have a mother who smoked during pregnancy [55, 56]. We also performed secondary analyses on the small subset of children with data on maternal smoking during pregnancy. It did not indicate substantial confounding in our ALL results, though we had limited power. Our inability to adjust for maternal smoking during pregnancy also may have resulted in residual confounding.

Because we did not have complete residential histories, we assumed that address at time of diagnosis represented a child’s residence over the entire exposure period, so residential mobility during the exposure period may have obscured our results. A California study on residential mobility in children with leukemia found that 66% of children moved between birth and diagnosis, urban/rural status changed for 20%, and neighborhood SES changed for 35% [57]. The lack of information on residence across the entire exposure period prior to diagnosis likely introduced exposure misclassification for both controls and cases, most likely biasing our estimates toward the null.

Using IDW well counts in absence of information on O&G well activities, meteorology, and topography likely reduced temporal and spatial specificity in IDW well counts [36]. The overall effect of the resulting exposure misclassification is unknown, but it would most likely have biased our associations toward the null [58, 59]. The subset of oil and gas wells for which a spud-in date was not available, and for which we instead used the earliest available date of activity, may have resulted in an underestimate of IDW well counts for both cases and controls, and thus may have biased our results towards the null [60]. However, this bias is likely minimal: in the 72% of wells with spud dates, the median number of days between spud date and the next well activity is 13 days.

## Conclusion

In this exploratory study, children aged 5–24 years diagnosed with ALL were more likely than children diagnosed with a non-hematologic cancer to live within 16.1-kilometers of an active oil and gas well, while children aged 0–4 years diagnosed with ALL were not more likely than children diagnosed with a non-hematologic cancer to live with within 16.1-kilometers of an active oil and gas well. Children aged 0–24 years diagnosed with NHL were no more likely to live in areas with active oil and gas development than children diagnosed with a non-hematologic cancer. Because oil and gas development has potential to expose a large population to known hematologic carcinogens, such as benzene, further study is clearly needed to substantiate both our positive and negative findings. Future studies should incorporate information on oil and gas development activities and production levels near homes, schools, and day care centers; provide age-specific residential histories; compare cases to controls without cancer; and address other potential confounders, and environmental stressors.

## Supporting information

S1 Table Location coordinates for active oil and gas wells by year. (ZIP)

S2 Table Adjusted logistic regression model 1 for association between annual inverse distance weighted well count within 16.1-kilometer radius of residence at diagnosis averaged over exposure period and acute lymphocytic leukemia (ALL). (PDF)

S3 Table Adjusted logistic regression model 2 for association between annual inverse distance weighted well count within 16.1-kilometer radius of residence at diagnosis averaged over exposure period and acute lymphocytic leukemia (ALL). (PDF)

S4 Table Association between acute lymphocytic leukemia (ALL) and annual inverse distance weighted well count within 8-kilometer radius of residence at diagnosis averaged over exposure period. (PDF)

S5 Table Association between acute lymphocytic leukemia (ALL) and annual inverse distance weighted well count within 16.1-kilometer radius of residence at diagnosis averaged over exposure period: Subset of subjects with information on maternal smoking. (PDF)

S6 Table Adjusted logistic regression model 1 for association between annual inverse distance weighted well count within 16.1-kilometer radius of residence at diagnosis averaged over exposure period and non-Hodgkin lymphoma. (PDF)

S7 Table Adjusted logistic regression model 2 for association between annual inverse distance weighted well count within 16.1-kilometer radius of residence at diagnosis averaged over exposure period and non-Hodgkin lymphoma. (PDF)

S8 Table Association between non-Hodgkin Lymphoma (NHL) and annual inverse distance weighted well count within 8-kilometer radius of residence at diagnosis averaged over exposure period total study population (0–24 years). (PDF)

S9 Table Association between non-Hodgkin Lymphoma (NHL) and annual inverse distance weighted well count within 16.1-kilometer radius of residence at diagnosis averaged over exposure period: Subset of subjects with information on maternal smoking total study population (0–24 years). (PDF)

S10 Table Association between annual inverse distance weighted well count within 16.1-kilometer radius of residence at diagnosis averaged over exposure period and acute lymphocytic leukemia (ALL): Each year of cancer diagnosis treated separately. (PDF)
