# Supplementary material for: Childhood hematologic cancer and residential proximity to oil and gas development
Source: PLoS One. 2017 Feb 15;12(2):e0170423. doi: 10.1371/journal.pone.0170423 (PMC5310851; doi:10.1371/journal.pone.0170423)
Supplement: S3 Table — (PDF) [file pone.0170423.s003.pdf]

Supplemental Material: Childhood Hematologic Cancer and Residential Proximity to Oil and Gas Development in Rural Colorado

Lisa M. McKenzie, William B. Allshouse, Tim E. Byers, Edward J. Bedrick, Berrin Serdar, and John L. Adgate

S3 Table: Adjusted logistic regression model 2 for association between annual inverse distance weighted well count within 16.1-kilometer radius of residence at diagnosis averaged over exposure period and acute lymphocytic leukemia (ALL)

S3 Table: Adjusted logistic regression model 2 for association between annual inverse distance weighted well count within 16.1-kilometer radius of residence at diagnosis averaged over exposure period and acute lymphocytic leukemia (ALL)

Odds Ratio Estimates			
**Effect**	**Point Estimate**	**95% Wald Confidence Limits**	
**Low Tertile[a]**	2.479	1.002	6.135
**Medium Tertile[a]**	2.794	1.152	6.776
**High Tertile[a]**	1.999	0.796	5.017
**White Hispanic[b]**	0.575	0.261	1.269
**Other race[b]**	1.600	0.671	3.817
**Female[c]**	0.789	0.467	1.331
**0-4 years[d]**	26.611	7.905	89.587
**5-9 years[d]**	42.430	11.876	151.589
**10 -14 years[d]**	12.679	3.386	47.476
**15-19 years[d]**	5.589	1.491	20.942
**≥9000 feet[e]**	3.536	0.947	13.199
**Zip code level income 21-40 percentile[f]**	0.791	0.290	2.156
**Zip code level income 41-60 percentile[f]**	0.754	0.248	2.295
**Zip code level income 61-80 percentile[f]**	0.859	0.310	2.384
**Zip code level income 81-100 percentile[f]**	0.974	0.355	2.677
**year 1 vs 0[g]**	1.493	0.565	3.946
**year 2 vs 0[g]**	1.525	0.557	4.178
**year 3 vs 0[g]**	1.850	0.700	4.885
**year 4 vs 0[g]**	0.856	0.315	2.327
**year 5 vs 0[g]**	1.402	0.549	3.581

[a] low = first tertile, greater than 0 to 2.7 wells per 1.6 kilometers, medium = second tertile, 2.7 to 31.4 wells per 1.6 kilometers, high = third tertile, more than 31.4 wells per 1.6 kilometers. [b]Reference group is white non-Hispanics. [c]Reference group is males. [d]Reference group is 20-24 years. [e]Reference group is < 9000 feet. [f]Reference group is 0-20 percentile. [g]Reference group is year 0.
