# Supplementary material for: Childhood hematologic cancer and residential proximity to oil and gas development
Source: PLoS One. 2017 Feb 15;12(2):e0170423. doi: 10.1371/journal.pone.0170423 (PMC5310851; doi:10.1371/journal.pone.0170423)
Supplement: S4 Table — (PDF) [file pone.0170423.s004.pdf]

Supplemental Material: Childhood Hematologic Cancer and Residential Proximity to Oil and Gas Development in Rural Colorado

Lisa M. McKenzie, William B. Allshouse, Tim E. Byers, Edward J. Bedrick, Berrin Serdar, and John L. Adgate

S4 Table: Association between acute lymphocytic leukemia (ALL) and annual inverse distance weighted well count within 8-kilometer radius of residence at diagnosis averaged over exposure period

S4 Table: Association between acute lymphocytic leukemia (ALL) and annual inverse distance weighted well count within 8-kilometer radius of residence at diagnosis averaged over exposure period.

**Inverse Distance Weighted Well Count**[a]	**0 Wells within 8 kilometers**	**Low**[a]	**Medium**[a]	**High**[a]	**P-value for trend test**[b]
Total Study Population (0 - 24 Years)					
Cases (N)	29	18	19	21	
Controls (N)	229	99	98	102	
Crude OR	1.0	1.4 (0.76, 2.7)	1.5 (0.82, 2.9)	1.6 (0.89, 3.0)	
Adjusted OR (95% CI)[c]	1.0	1.7 (0.81, 3.5)	1.7 (0.83, 3.5)	1.6 (0.76, 3.4)	0.16
5 to 24 Years					
Cases (N)	11	10	15	12	
Controls (N)	190	84	81	76	
Crude OR	1.0	2.1 (0.84, 5.0)	3.2 (1.4, 7.3)	2.7 (1.2, 6.4)	
Model 1 Adjusted OR (95% CI)[c]	1.0	2.7 (0.92, 8.2)	4.0 (1.4, 11)	3.8 (1.2, 12)	0.0094
0-4 Years					
Cases (N)	18	8	< 5	9	
Controls (N)	39	15	17	26	
Crude OR	1.0	1.2 (0.42, 3.2)	0.51 (0.15, 1.7)	0.75 (0.29, 1.9)	
Adjusted OR (95% CI)[d]	1.0	1.4 (0.46, 4.4)	0.47 (0.12, 1.7)	0.76 (0.26, 2.2)	0.36

[a] low = first tertile, greater than 0 to 2.7 wells per 1.6 kilometers, medium = second tertile, 2.7 to 31.4 wells per 1.6 kilometers, high = third tertile, more than 31.4 wells per 1.6 kilometers. [b]Trend testsperformed by treating categorical inverse-distance well count as an ordinal. [c]Adjusted for age, race, gender, zip code level median income, and elevation. [d]Adjusted for race, gender, zip code level median income, and elevation.
