# Supplementary material for: Childhood hematologic cancer and residential proximity to oil and gas development
Source: PLoS One. 2017 Feb 15;12(2):e0170423. doi: 10.1371/journal.pone.0170423 (PMC5310851; doi:10.1371/journal.pone.0170423)
Supplement: S5 Table — (PDF) [file pone.0170423.s005.pdf]

Supplemental Material: Childhood Hematologic Cancer and Residential Proximity to Oil and Gas Development in Rural Colorado

Lisa M. McKenzie, William B. Allshouse, Tim E. Byers, Edward J. Bedrick, Berrin Serdar, and John L. Adgate

S5 Table: Association between acute lymphocytic leukemia (ALL) and annual inverse distance weighted well count within 16.1-kilometer radius of residence at diagnosis averaged over exposure period : Subset of subjects with information on maternal smoking

S5 Table: Association between acute lymphocytic leukemia (ALL) and annual inverse distance weighted well count within 16.1-kilometer radius of residence at diagnosis averaged over exposure period: Subset of subjects with information on maternal smoking.

**Inverse Distance Weighted Well Count[a]**	**0 Wells within 8 kilometers**	**Low[a]**	**Medium[a]**	**High[a]**	**P-value for trend test[b]**
(Total Study Population) 0 - 24 Years					
Cases (N)	8	11	15	18	
Controls (N)	58	39	55	58	
Crude OR	1.0	2.0 (0.75, 5.4)	2.0 (0.78, 5.0)	2.3 (0.91, 5.6)	
Adjusted OR (95% CI)[c]	1.0	2.2 (0.64, 7.3)	2.0 (0.62, 6.2)	1.5 (0.47, 4.8)	0.72
5 to 24 Years					
Cases (N)	<5	<5	6	10	
Controls (N)	44	22	37	28	
Crude OR	1.0	1.3 (0.21, 8.6)	2.4 (0.56, 10)	5.2 (1.3, 21)	
Model 1 Adjusted OR (95% CI)[c]	1.0	2.0 (0.20, 21)	2.4 (0.36, 16)	2.9 (0.42, 20)	0.29
0-4 Years					
Cases (N)	5	9	9	8	
Controls (N)	14	17	18	30	
Crude OR	1.0	1.5 (0.40, 5.5)	1.4 (0.38, 5.1)	0.75 (0.21, 2.7)	
Adjusted OR (95% CI)[d]	1.0	1.5 (0.31, 7.3)	1.5 (0.30, 7.1)	0.77 (0.16, 3.8)	0.56

[a] Low = first tertile, < 4.96 wells per 1.6 kilometers, medium = second tertile, 4.9 to 33.6 wells per 1.6 kilometers, high = third tertile, more than 33.6 wells per 1.6 kilometers. [b]Trend testsperformed by treating categorical inverse-distance well count as an ordinal.. [c]Adjusted for age, race, gender, zip code level median income, elevation, and maternal smoking. [d]Adjusted for race, gender, elevation, zip code level median income, and maternal smoking.
