# Supplementary material for: Childhood hematologic cancer and residential proximity to oil and gas development
Source: PLoS One. 2017 Feb 15;12(2):e0170423. doi: 10.1371/journal.pone.0170423 (PMC5310851; doi:10.1371/journal.pone.0170423)
Supplement: S7 Table — (PDF) [file pone.0170423.s007.pdf]

Supplemental Material: Childhood Hematologic Cancer and Residential Proximity to Oil and Gas Development in Rural Colorado

Lisa M. McKenzie, William B. Allshouse, Tim E. Byers, Edward J. Bedrick, Berrin Serdar, and John L. Adgate

S7 Table: Adjusted logistic regression model 2 for association between annual inverse distance weighted well count within 16.1-kilometer radius of residence at diagnosis averaged over exposure period and non-Hodgkin lymphoma

S7 Table: Adjusted logistic regression model 2 for association between annual inverse distance weighted well count within 16.1-kilometer radius of residence at diagnosis averaged over exposure period and non-Hodgkin lymphoma

Odds Ratio Estimates			
**Effect**	**Point Estimate**	**95% Wald Confidence Limits**	
**Low Tertile[a]**	1.177	0.487	2.844
**Medium Tertile[a]**	0.632	0.247	1.620
**High Tertile[a]**	0.985	0.389	2.494
**White Hispanic[b]**	0.431	0.126	1.482
**Other race[b]**	2.615	1.038	6.590
**Female[c]**	0.487	0.252	0.943
**0-4 years[d]**	0.504	0.169	1.503
**5-9 years[d]**	3.177	1.213	8.324
**10 -14 years[d]**	1.912	0.770	4.748
**15-19 years[d]**	1.081	0.472	2.477
**≥9000 feet[e]**	0.570	0.063	5.186
**Zip code level income 21-40 percentile[f]**	2.141	0.422	10.872
**Zip code level income 41-60 percentile[f]**	8.438	1.615	44.081
**Zip code level income 61-80 percentile[f]**	3.728	0.743	18.691
**Zip code level income 81-100 percentile[f]**	2.517	0.475	13.330
**year 1 vs 0[g]**	1.655	0.525	5.221
**year 2 vs 0[g]**	1.509	0.428	5.321
**year 3 vs 0[g]**	0.846	0.234	3.058
**year 4 vs 0[g]**	1.815	0.585	5.633

Odds Ratio Estimates			
**Effect**	**Point Estimate**	**95% Wald Confidence Limits**	
**year 5 vs 0[g]**	0.876	0.266	2.886

[a] low = first tertile, greater than 0 to 2.7 wells per 1.6 kilometers, medium = second tertile, 2.7 to 31.4 wells per 1.6 kilometers, high = third tertile, more than 31.4 wells per 1.6 kilometers. [b]Reference group is white non-Hispanics. [c]Reference group is males. [d]Reference group is 20-24 years. [e]Reference group is < 9000 feet. [f]Reference group is 0-20 percentile. [g]Reference group is year 0.
