# Supplementary material for: Childhood hematologic cancer and residential proximity to oil and gas development
Source: PLoS One. 2017 Feb 15;12(2):e0170423. doi: 10.1371/journal.pone.0170423 (PMC5310851; doi:10.1371/journal.pone.0170423)
Supplement: S8 Table — (PDF) [file pone.0170423.s008.pdf]

Supplemental Material: Childhood Hematologic Cancer and Residential Proximity to Oil and Gas Development in Rural Colorado

Lisa M. McKenzie, William B. Allshouse, Tim E. Byers, Edward J. Bedrick, Berrin Serdar, and John L. Adgate

S8 Table: Association between non-Hodgkin Lymphoma (NHL) and annual inverse distance weighted well count within 8-kilometer radius of residence at diagnosis averaged over exposure period total study population (0-24 years)

S8 Table: Association non-Hodgkin Lymphoma (NHL) and between annual inverse distance weighted well count within 8-kilometer radius of residence at diagnosis averaged over exposure period total study population (0-24 years)

**Inverse Distance Weighted Well Count**	**0 Wells within 8 kilometers**	**Low[a]**	**Medium[a]**	**High[a]**	**P-value for trend test[b]**
Cases (N)	18	12	7	13	
Controls (N)	229	99	98	102	
Crude OR	1.0	1.5 (0.72, 3.3)	0.91 (0.37, 2.2)	1.6 (0.77, 3.4)	
Model 1Adjusted OR (95% CI)[c]	1.0	1.4 (0.62, 3.2)	0.66 (0.25, 1.7)	1.7 (0.71, 3.9)	0.56

[a] low = first tertile, greater than 0 to 2.7 wells per 1.6 kilometers, medium = second tertile, 2.71 to 31.4 wells per 1.6 kilometers, high = third tertile, 31.5 or more wells per 1.6 kilometers. [b]Trend testsperformed by treating categorical inverse-distance well count as an ordinal. [c]Adjusted for age, race, gender, zip code level median income, and elevation.
