# Supplementary material for: Childhood hematologic cancer and residential proximity to oil and gas development
Source: PLoS One. 2017 Feb 15;12(2):e0170423. doi: 10.1371/journal.pone.0170423 (PMC5310851; doi:10.1371/journal.pone.0170423)
Supplement: S9 Table — (PDF) [file pone.0170423.s009.pdf]

Supplemental Material: Childhood Hematologic Cancer and Residential Proximity to Oil and Gas Development in Rural Colorado

Lisa M. McKenzie, William B. Allshouse, Tim E. Byers, Edward J. Bedrick, Berrin Serdar, and John L. Adgate

S9 Table: Association between non-Hodgkin Lymphoma (NHL) and annual inverse distance weighted well count within 16.1-kilometer radius of residence at diagnosis averaged over exposure period : Subset of subjects with information on maternal smoking total study population (0-24 years)

S9 Table. Association between non-Hodgkin lymphoma (NHL) and annual inverse distance weighted well count within 16.1-kilometer radius of residence at diagnosis averaged over exposure period: Subset of subjects with information on maternal smoking total study population (0-24 years)

**Inverse Distance Weighted Well Count**	**0 Wells within 8 kilometers**	**Low**[a]	**Medium**[a]	**High**[a]	**P-value for trend test**[b]
Cases (N)	<5	6	6	7	
Controls (N)	58	39	55	58	
Crude OR	1.0	3.0 (0.70, 13)	2.1 (0.50, 8.9)	2.3 (0.58, 9.5)	
Model 1Adjusted OR (95% CI)[c]	1.0	4.7 (0.86, 26)	1.5 (0.28, 7.7)	1.8 (0.33, 9.3)	0.97

[a] low = first tertile, < 4.9 wells per 1.6 kilometers, medium = second tertile, 4.9 to 33.6 wells per 1.6 kilometers, high = third tertile, more than 33.6 wells per 1.6 kilometers.

[b]Trend testsperformed by treating categorical inverse-distance well count as an ordinal. [c]Adjusted for age, race, gender, elevation, zip code level median income, and maternal smoking.
