# Supplementary material for: Childhood hematologic cancer and residential proximity to oil and gas development
Source: PLoS One. 2017 Feb 15;12(2):e0170423. doi: 10.1371/journal.pone.0170423 (PMC5310851; doi:10.1371/journal.pone.0170423)
Supplement: S10 Table — (PDF) [file pone.0170423.s010.pdf]

# Supplemental Material: Childhood Hematologic Cancer and Residential Proximity to Oil and Gas Development in Rural Colorado

Lisa M. McKenzie, William B. Allshouse, Tim E. Byers, Edward J. Bedrick, Berrin Serdar, and John L. Adgate

**S10 Table.** Association between annual inverse distance weighted well count within 16.1-kilometer radius of residence at diagnosis averaged over exposure period and acute lymphocytic leukemia (ALL): Each year of cancer diagnosis treated separately

**S10 Table. Association between annual inverse distance weighted well count within 16.1-kilometer radius of residence at diagnosis averaged over exposure period and acute lymphocytic leukemia (ALL): Each year of cancer diagnosis treated separately.**

| Inverse Distance Weighted Well Count[a] | 0 Wells within 16.1 Kilometers | Low[a] | Medium[a] | High[a] | P-value trend tests[b] |
|---|---|---|---|---|---|
| ***Total Study Population (0 to 24 years)*** | | | | | |
| Cases (N) | 15 (9.2%) | 21 (14%) | 26 (18%) | 25 (16%) | |
| Controls (N) | 147 | 132 | 119 | 130 | |
| Crude OR | 1.0 | 1.6 (0.77, 3.1) | 2.1 (1.1, 4.2) | 1.9 (0.95, 3.7) | |
| Model 1 Adjusted OR (95% CI)[c] | 1.0 | 2.3 (0.94, 5.5) | 2.6 (1.1, 6.3) | 1.9 (0.78, 4.8) | 0.22 |
| Model 2 Adjusted OR (95% CI)[d] | 1.0 | 2.4 (0.97, 6.1) | 2.9 (1.2, 7.1) | 1.9 (0.76, 4.9) | 0.23 |
| ***5 to 24 Years*** | | | | | |
| Cases (N) | 8 (5.9%) | 9 (7.7%) | 15 (13%) | 16 (14%) | |
| Controls (N) | 128 | 108 | 99 | 96 | |
| Crude OR | 1.0 | 1.3 (0.50, 3.6) | 2.4 (0.99, 5.9) | 2.7 (1.1, 6.5) | |
| Model 1 Adjusted OR (95% CI)[c] | 1.0 | 2.9 (0.80, 11) | 3.4 (0.99, 12) | 4.3 (1.1, 16) | 0.035 |
| Model 2 Adjusted OR (95% CI)[d] | 1.0 | 2.6 (0.67, 10) | 3.6 (1.0, 13) | 3.6 (0.93, 14) | 0.056 |
| ***0 to 4 Years*** | | | | | |
| Cases (N) | 7 (26%) | 12 (33%) | 11 (35%) | 9 (21%) | |
| Controls (N) | 19 | 24 | 20 | 34 | |
| Crude OR | 1.0 | 1.4 (0.45, 4.1) | 1.5 (0.48, 4.7) | 0.72 (0.23, 2.2) | |
| Model 1Adjusted OR (95% CI)[e] | 1.0 | 1.7 (0.44, 6.5) | 2.3 (0.57, 9.4) | 0.73 (0.18, 3.0) | 0.50 |
| Model 2 Adjusted OR (95% CI)[f] | 1.0 | 1.7 (0.42, 7.2) | 2.5 (0.56, 12) | 0.5 (0.11, 2.3) | 0.25 |

[a] All age groups: low = first tertile, < 4.9 wells per 1.6 kilometers, medium = second tertile, 4.9 to 33.6 wells per 1.6 kilometers, high = third tertile, more than 33.6 wells per 1.6 kilometers. [b]Trend tests performed by treating categorical inverse-distance well count as an ordinal. [c]Adjusted for age, race, gender, socioeconomic status, and elevation. [d]Adjusted for age, race, gender, socioeconomic status, elevation, and year of diagnosis. [e]Adjusted for race, gender, socioeconomic status, and elevation. [f]Adjusted for race, gender, socioeconomic status, elevation, and year of diagnosis.
